# Pediatric fatal head and neck injury in a 9-year medical examiner case series: autopsy findings and cervical spine dissection techniques

**DOI:** 10.1007/s00414-026-03839-5

**Published:** 2026-05-29

**Authors:** Enrica Macorano, Francesco Introna, Carlo Pietro Campobasso, Lorenzo Gitto

**Affiliations:** 1 Section of Legal Medicine, Department of Interdisciplinary Medicine, University of Bari “Aldo Moro”, Bari Policlinico Hospital, Bari, 70124 Italy; 2 Department of Experimental Medicine, University of Campania “Luigi Vanvitelli”, via Luciano Armanni 5, Naples, 80138 Italy; 3 Department of Pathology, Cook County Medical Examiner’s Office, 2121 W Harrison Street, Chicago, IL 60612 USA; 4 Department of Mental and Physical Health and Preventive Medicine, University of Campania “Luigi Vanvitelli”, 80138 Naples, Italy

**Keywords:** Child abuse, Pediatric non-accidental head trauma, Cervical spine dissection, Autopsy, Forensic pathology

## Abstract

**Background:**

Fatal inflicted head injury in infants requires systematic postmortem evaluation of intracranial, ocular, and cervicomedullary structures. This study summarizes autopsy findings in homicidal blunt head trauma in infants under one year of age and describes an en bloc cervical spine dissection approach intended to improve assessment of the cranio cervical region.

**Methods:**

A retrospective review was performed at the Cook County Medical Examiner’s Office, Chicago, Illinois, USA, covering 2015 to 2023. Included cases were infants under one year of age with homicide certification and blunt force head injury. Autopsy reports, radiology, neuropathology, and ophthalmology were reviewed, with investigative and child welfare records when available. Injuries were mainly categorized across cranial, spinal, and ocular.

**Results:**

Sixty one infant homicides were identified; thirty nine met inclusion criteria. Acute subdural hemorrhage and subarachnoid hemorrhage were each present in 35 of 39 cases (89.7%). Retinal hemorrhages occurred in 34 of 39 (87.2%), with bilateral involvement in 31 (79.5%). Perineural optic nerve subdural hemorrhage was present in 33 (84.6%), bilateral in 32 (82.1%). Skull fractures were identified in 18 (46.2%). Cervical spinal cord injury was documented in 16 (41.1%), most commonly hemorrhagic. Remote extracranial fractures were present in 26 (66.7%), and recent extracranial fractures in 16 (41.1%).

**Conclusion:**

Intracranial and ocular hemorrhages were frequent in this large medical examiner series, and cervical spinal cord injury was common. The findings support targeted evaluation of the cervical spine in selected infant homicide cases, with en bloc techniques as a useful option when resources and expertise permit.

## Introduction

Fatal head injury in infants and young children represents a critical area of forensic investigation, requiring meticulous postmortem examination and objective documentation of traumatic findings. The forensic pathologist plays a central role in identifying, describing, and interpreting injuries of the central nervous system and associated structures, particularly when deaths are referred under circumstances raising concern for inflicted trauma.

Early descriptions of inflicted injury, including physical abuse and infanticide, emphasized the importance of careful postmortem evaluation in distinguishing traumatic findings from natural disease or accidental injury [1, 2]. The publication of “The Battered Child Syndrome” by Henry Kempe in the 1960s marked a pivotal moment in highlighting the diagnostic value of detailed injury assessment, particularly in fatal cases involving young children [3]. These developments contributed to the establishment of coordinated medico-legal frameworks, including the Child Abuse Prevention and Treatment Act (CAPTA) of 1974 in the United States, which standardized investigative approaches across healthcare and legal systems [4].

Within this evolving context, injuries involving the head and brain emerged as the most frequent and consequential findings in fatal pediatric cases referred for forensic investigation. During the 1970s, the term shaken baby syndrome was introduced to describe characteristic intracranial and ocular findings observed in infants with fatal or severe head injury in the absence of prominent external trauma [5, 6]. These observations were classically summarized as a frequently reported combination of subdural hemorrhage, retinal hemorrhages, and hypoxic-ischemic encephalopathy, with proposed diagnostic criteria based on autopsy and clinical correlations [7–9]. When skull fractures or focal intracranial hemorrhages were present, alternative terminology was used to describe head injury associated with direct impact.

As understanding evolved, emphasis shifted toward descriptive terminology that encompassed a range of traumatic findings without reliance on a single inferred mechanism. In 2009, the American Academy of Pediatrics introduced the term abusive head trauma to describe inflicted head injuries in infants and children, encompassing intracranial findings such as subdural hematomas, retinal hemorrhages, skull fractures, and diffuse axonal injury (DAI) [10]. More recently, additional descriptive entities, including the so-called “Compressed Baby Head”, have been proposed based on specific constellations of cranial findings observed at autopsy [11].

Numerous studies have examined injury distributions, autopsy findings, and proposed mechanisms in pediatric fatal head injury cases investigated under circumstances of suspected maltreatment [12–14]. Although biomechanical interpretations remain debated, head injury remains the leading cause of death in children investigated for physical abuse [15, 16] and may be associated with injuries to the cervical spine and associated nerve roots that can be incompletely assessed with routine autopsy techniques.

The present study retrospectively reviews autopsy findings in fatal pediatric head injury cases involving individuals under one year of age examined at a large metropolitan medical examiner’s office over a nine-year period, with particular attention to intracranial, cervical spinal cord, and associated soft tissue findings. The observed findings are compared with existing literature. In addition, based on recurrent anatomic observations, an alternative en bloc cervical spine dissection technique is described as a technical approach to enhance postmortem evaluation of the cranio-cervical region in pediatric fatal head injury cases.

## Methods

A retrospective review of pediatric cases from the Cook County Medical Examiner’s Office in Chicago, Illinois, United States (CCMEO) was conducted using the office digital database ‘LabLynx’ (LabLynx, Inc., Smyrna, Georgia, U.S). Cases of children under one year of age in whom the manner of death was certified as homicide and in whom blunt force head injuries were found were selected. In each case, autopsy reports, toxicology findings, investigative and police reports, and, when available, reports from the Department of Children and Family Services were systematically reviewed alongside radiological studies, neuropathological examinations, and ophthalmological assessments; toxicology, investigative, police and Department of Children and Family Services reports, when available, were reviewed.

All autopsies were performed by board-certified forensic pathologists at the Cook County Medical Examiner’s Office. Neuropathological and ophthalmological evaluations were conducted by board-certified specialists affiliated with local academic institutions. Brain, spinal cord, and ocular tissue removal and examination followed standard autopsy protocols as outlined in the literature [17–23].

Following case selection, documented injuries were categorized based on external and internal findings. Radiological studies, histological findings, neuropathological and ophthalmological examinations, and toxicological results were reviewed to identify and correlate additional injury patterns and relevant ancillary data.

Data was organized using Microsoft Excel (Microsoft Corporation, Redmond, Washington, USA) with emphasis on the following variables: sample size, age, sex, race, scalp and facial trauma, subgaleal hemorrhage (SGH), skull fractures, extradural, intradural, and subdural hemorrhages (EDH, IDH, and SDH), subarachnoid hemorrhage (SAH), intracerebral and spinal cord pathology, and ocular findings.

## Results

### Study population

Sixty-one cases of pediatric homicide involving subjects under 1 year of age were found in the studied period. Within this cohort, 39 met the study criteria described above (*n* = 39). Cases excluded from analysis (*n* = 22) involved homicides attributed to other causes, including drug toxicity, gunshot wounds, complications of prematurity, hypoxic-ischemic encephalopathy, drowning, and scald injuries.

For age-based analysis, the study population was divided into two groups: neonates (< 1 month, *n* = 2) and infants (1–12 months, *n* = 37). The sex distribution consisted of 20 females and 19 males. Racial demographics included 24 African American, 13 Caucasian, and 2 individuals of other racial backgrounds.

A summary of the external and internal findings is provided in Table 1. The findings reported in the table were sometimes observed in isolation and sometimes in combination, coexisting in the same individual.

**Table 1:** Summary of the pathologic findings (*n* = 39). [*EDH*,* extradural hemorrhage; IDH*,* intradural hem.; SDH*,* subdural hem.; SAH*,* subarachnoid hem. Perc.*,* percentage.*]

FINDINGS		All (*n* = 39)	Perc (%)
REMOTE INJURIES			
Head external injuries	No	36	92,3
	Any	3	7,6
Body external injuries	No	33	84,6
	Yes	6	15,4
Extracranial fractures	No	26	66,7
	Yes	13	33,3
CRANIAL INJURIES			
Scalp external injuries	No	22	56,4
	Yes	17	43,6
Face external injuries	No	19	48,7
	Any	20	51,3
Subgaleal hemorrhages	No	23	58,9
	Yes	16	41,1
Skull fractures	No	21	53,8
	Yes	18	46,2
Suture diastasis	No	33	84,6
	Any	6	15,4
EDH	No	34	87,2
	Yes	5	12,8
Acute SDH	No	4	10,3
	Yes	35	89,7
Older SDH	No	32	82,1
	Yes	7	17,9
Overall SDH	No	34	87,2
	Any	5	12,8
IDH	No	36	92,3
	Yes	3	7,7
SAH	No	4	10,3
	Yes	35	89,7
Intracerebral hemorrhage	No	19	48,7
	Yes	20	51,3
Intracerebral necrosis	No	27	69,2
	Yes	12	30,8
Global hypoxia ischemia	No	8	20,5
	Any	31	79,5
Intracerebral findings	No	27	69,2
	Any	12	30,8
Cerebral hemispheres findings	No	6	15,4
	Any	33	84,6
Cerebellum findings	No	20	51,3
	Any	19	48,7
Brainstem findings	No	25	64,1
	Any	14	35,9
Cerebral edema	No	15	38,5
	Yes	24	61,5
Diffuse Axonal Injury (DAI)	No	32	82,1
	Yes	7	17,9
Basal ganglia findings	No	27	69,2
	Any	12	30,8
SPINAL CORD INJURIES			
Cervical spinal cord injuries	No	23	58,9
	Any	16	41,1
Cervical spinal cord EDH	No	22	56,4
	Any	17	43,6
Cervical spinal cord SDH	No	25	64,1
	Any	14	35,9
Cervical spinal cord SAH	No	34	87,2
	Any	5	12,8
Cervical spinal cord Int hem	No	33	84,6
	Any	6	15,4
Cervical spinal cord Necrosis	No	37	94,9
	Any	2	5,1
OCULAR INJURIES			
Retinal hemorrhages	No	5	12,8
	Any	34	87,2
Bilateral retinal hemorrhages	No	8	20,5
	Yes	31	79,5
Macular fold	No	38	97,4
	Any	1	2,6
Vitreous hemorrhages	No	36	92,3
	Any	3	7,7
Bilateral vitreous hemorrhages	No	37	94,9
	Yes	2	5,1
Optic nerve perineural SDH	No	6	15,4
	Any	33	84,6
Bilateral optic nerve perineural SDH	No	7	17,9
	Yes	32	82,1

### Remote injuries

**Fig. 1:**
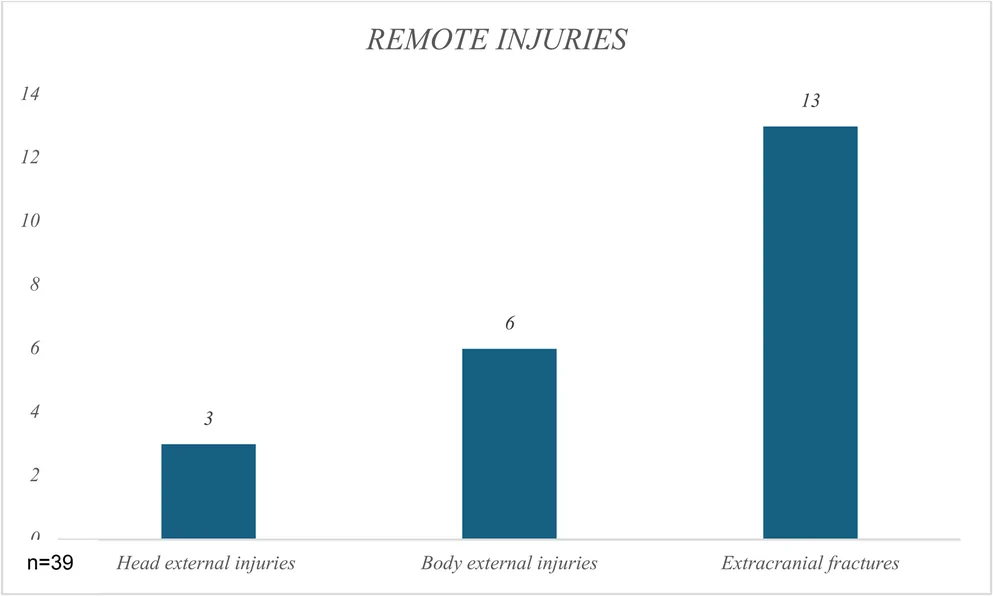
Prevalence of remote injuries observed in the study population (*n* = 39)

Evidence of remote (subacute or healed) extracranial fractures (Fig. 1) was identified in 66.7% of cases (*n* = 26). Among these, rib fractures were observed in 11 cases, femur fractures in 2, tibia fractures in 2, radius fractures in 2, an ulna fracture in 1, and a vertebral fracture in 1.

External injuries, including contusions and abrasions, were noted on the head in 7.7% of cases (*n* = 3) and on the body in 15.4% of cases (*n* = 6).

### Skull and brain pathology

**Fig. 2:**
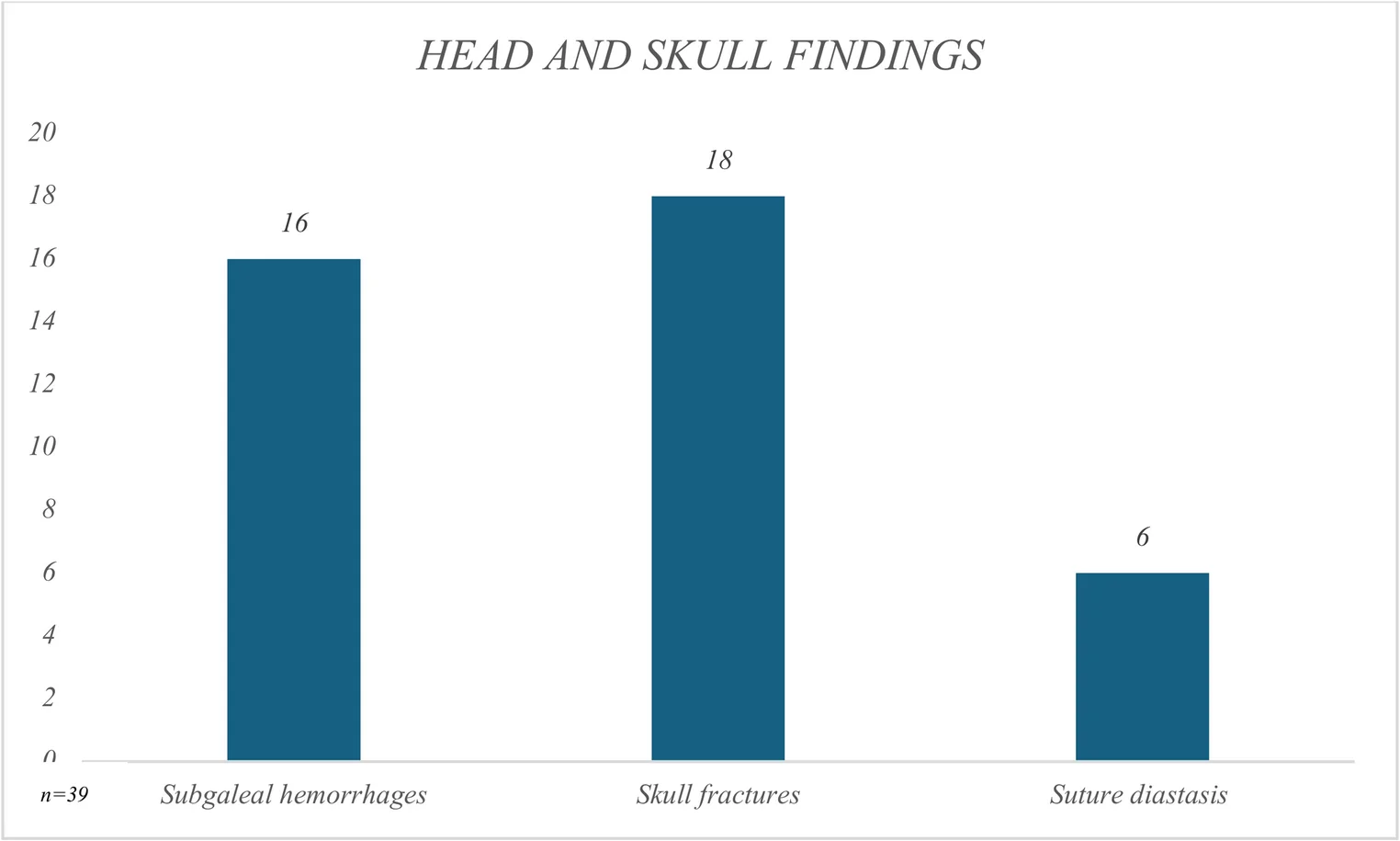
Prevalence of “head and skull findings” in the study population (*n* = 39)

Skull fractures (Fig. 2) were identified in 46.15% of cases (*n* = 18), most commonly involving the parietal bones, with additional involvement of the occipital and, less frequently, the frontal bones. The majority of fractures exhibited a linear configuration. Several cases demonstrated bilateral fractures, including one instance with bilateral and multiple fractures: one case featured complex fractures of both the left and right parietal bones; another involved bilateral fracture of the occipital and parietal bones extending into the bilateral posterior cranial fossae. Overall, three cases exhibited bilateral fractures, and two cases involved multiple fracture lines.

In 15 of the 18 cases with skull fractures, no associated extradural hemorrhage was identified; however, other intracranial injuries were present. Diastasis of the cranial sutures was observed in 15.4% of cases (*n* = 6).

Brain findings revealed extradural hemorrhages (EDH) in 12.8% of cases (*n* = 5). Intradural hemorrhage (IDH) was identified in 7.7% of cases (*n* = 3), with one case described as “focal.”

Subdural hemorrhage (SDH) was the most prevalent intracranial injury, observed in 92.3% of cases (*n* = 36). Of these, 35 were acute SDH, including 7 cases with bilateral hemispheric involvement. Additionally, 7 cases demonstrated older subdural hemorrhages described as “old,” “organized,” or “organizing,” with six occurring alongside acute SDH and one in isolation.

Subarachnoid hemorrhage (SAH) was present in 35 of 39 cases. All SAHs involved the cerebral convexities; 13 were bilateral, 7 diffuse, and the remainder focal.

Cerebral edema was noted in 61.5% of subjects (*n* = 24). The most frequent microscopic finding was global hypoxic–ischemic injury, present in 79.5% of cases (*n* = 31), followed by focal hemorrhages in 51.3% (*n* = 20) and necrosis in 30.8% (*n* = 12). Diffuse axonal injury (DAI) was detected in seven cases; however, β-APP (beta-amyloid precursor protein) immunohistochemistry was not routinely performed during the study period.

Brain findings were the most frequently observed (84.6%, *n* = 33), followed by cerebellar findings (48.7%, *n* = 19) and brainstem findings (35.9%, *n* = 14). In addition, histologic examination identified basal ganglia injury in 12 of the 39 cases analyzed (30.8%). Furthermore, autopsy reports documented evidence of intracranial mass effect with bulging fontanelles in 12 of the 39 cases.

### Spinal cord neuropathology

Spinal cord injuries were identified in 41% of cases (*n* = 16) within the cervical region, while 30.8% (*n* = 12) and 20.5% (*n* = 8) were in the thoracic and lumbosacral regions respectively. The predominant finding was a thin layer of extradural hemorrhage (EDH), observed in 26 cases involving the cervical spinal cord. Within the cervical spinal cord, additional injuries included 14 cases of subdural hemorrhage (SDH), 5 cases of subarachnoid hemorrhage (SAH), 6 cases of intraparenchymal hemorrhage, and 2 cases demonstrating necrosis.

### Ocular pathology

Ocular pathology revealed that retinal hemorrhages and perineural optic nerve subdural hemorrhages (SDH) were frequent findings. Retinal hemorrhages were observed in 87.2% of subjects (*n* = 34), with 33 cases showing bilateral involvement. Perineural optic nerve SDH was present in 84.6% of cases (*n* = 33), with 32 exhibiting bilateral distribution. All retinal hemorrhages involved the intraretinal layer. A significant correlation was noted between intracranial subdural hemorrhage and both retinal hemorrhages and perineural optic nerve SDH. Vitreous hemorrhages were less common, identified in 7.7% of cases (*n* = 3), including two bilateral occurrences. Macular folds were rare, documented in only one case.

## Discussion

This study presents a descriptive analysis of postmortem findings observed in infants under one year of age with homicidal blunt head trauma examined at a large metropolitan medical examiner’s office. The aim was not to define diagnostic injury patterns or to infer mechanisms of injury, but to document the distribution and frequency of intracranial, ocular, spinal, and extracranial findings encountered in this case series and to discuss technical considerations relevant to their postmortem assessment.

Intracranial injuries were common in this cohort, consistent with prior forensic and clinical reports describing infant head trauma [24, 25]. The present findings are limited to anatomical distribution and histopathological observations and do not address mechanism, timing, or force thresholds. Skull fractures were present in 46.2% of cases, a prevalence comparable to that reported in other case series of AHT [26–28]. Brain injuries are commonly associated with progressive deterioration of consciousness due to increased intracranial pressure, which may clinically manifest as bulging of the fontanelles [29]. The relatively low frequency of epidural hemorrhage (EDH) observed in this series (12.8%) is consistent with the literature, as in young children the dura is more firmly adherent to the inner table of the skull, the middle meningeal artery is not yet fully embedded within a bony groove, and the calvarial bones are more pliable, all of which reduce the likelihood of meningeal vessel laceration following blunt impact [30]. In contrast, acute subdural hemorrhage (SDH) and subarachnoid hemorrhage (SAH) were frequently identified (both 89.7%). In infants, SDH often presents as a thin film and has been attributed, in prior biomechanical and autopsy studies, to rupture of bridging veins associated with rotational acceleration–deceleration forces [31–33].

Diffuse axonal injury was identified in a subset of cases. The absence of routine immunohistochemical staining during the study period represents a methodological limitation and may have contributed to underrecognition [34, 35].

Cervical spinal cord injury was identified in 41.1% of cases. These injuries, primarily EDH, underscore the vulnerability of the pediatric cervical spine, attributed to anatomical features such as ligamentous laxity and relatively large head size [36–38].

The absence of vertebral fractures is consistent with existing studies indicating that spinal injuries in infants are typically hemorrhagic or ligamentous rather than osseous in nature [39].

The co-occurrence of injuries, such as SDH, retinal hemorrhages, and rib fractures, is a combination of injuries reported in prior forensic series and concerning for inflicted trauma [12]. Published data report extremely low fatality rates from short-distance falls, which rarely produce diffuse intracranial or retinal hemorrhages [40–42].

Ocular findings are commonly reported in pediatric head trauma. In this study, retinal hemorrhages (87.2%) and optic nerve sheath hemorrhages (84.6%) were frequently observed, consistent with findings described in prior forensic and clinical literature [43–45]. Several mechanisms have been proposed to account for these findings, including acute increases in intracranial pressure, vitreoretinal traction, and rupture of the central retinal vein [46].

Retinal hemorrhages, particularly when bilateral or multilayered, have been reported less frequently in accidental trauma and more often in cases involving severe head injury. Prior biomechanical and clinical studies have proposed angular acceleration deceleration forces, including shaking or impact, as possible contributors to these findings, although they are not pathognomonic. Consensus statements and professional guidelines have noted that minor falls or routine activities are generally not associated with the extent or patterns of retinal hemorrhage described in severe infant head trauma [47]. Although uncommon, retinal hemorrhages related to cardiopulmonary resuscitation cannot be excluded a priori and, when present, are typically few, intraretinal, and confined to the posterior pole [48]. When available, premortem ophthalmologic examination records should be reviewed to complement postmortem findings [49].

The interpretation of ocular findings should be considered within the broader context of associated injuries. Prior work has suggested that the number and combination of findings, including intracranial lesions with retinal hemorrhages or skeletal injuries, may contribute to assessment of severe head trauma [50]. In the present series, multiple injury types were frequently observed within the same individuals, underscoring the need for a comprehensive and integrated postmortem evaluation.

Accordingly, pediatric forensic autopsy in infants requires particular care due to anatomical fragility and the wide differential diagnosis among natural, accidental, and violent causes of death. Pre autopsy imaging represents an essential component of the evaluation, with standardized radiological protocols recommended in suspected abusive head trauma cases [17, 51, 52]. When available, computed tomography and magnetic resonance imaging provide the greatest diagnostic yield, while conventional radiographic skeletal surveys remain essential for the detection of skeletal trauma.

A thorough external examination is mandatory. All skin lesions, including bruises, abrasions, burns, and bite marks, should be carefully documented and photographed, with particular attention to findings that are unusual or inconsistent with accidental trauma, especially in non-ambulatory infants [53].

Standard autopsy in infants includes a Y shaped incision with complete examination of the scalp, skull, and brain. Key findings include subgaleal hemorrhage, cranial sutures diastasis, fontanelle tension, and intracranial and retinal hemorrhages [54].

Given the importance of accurately documenting intraocular hemorrhage in cases of suspected infant and young child head trauma, removal of the eyes with a portion of each optic nerve and submission for ophthalmopathologic examination is recommended. The eyes and optic nerves can be easily removed with a posterior approach from the intracranial cavity to preserve ocular structures [17–19]. A layered anterior neck dissection is recommended to assess hemorrhage around the hyoid bone and adjacent soft tissues, and the thoracoabdominal examination should document costal fractures and visceral injuries.

In selected cases, deeper dissection of subcutaneous tissues and musculature may reveal occult hemorrhage. Cerebrospinal fluid collection may be performed for ancillary studies with caution to avoid anatomic disruption [17–19]. Vitreous humor should not be collected in cases of obvious head trauma, as the eyes require removal for ophthalmopathologic evaluation.

While standard and selective ancillary procedures may identify additional injuries, certain anatomical regions, particularly the cervical spine and associated nerve roots, may remain incompletely assessed without targeted dissection. In this context, prior research has reported an association between suspected neck hyperflexion–extension and unilateral or bilateral hemorrhages involving the cervical spinal nerve roots, most consistently at levels C3–C5, whereas such findings were uncommon in cases without suspected neck injury [55]. The authors proposed that injury to these nerve roots, which innervate the diaphragm, could contribute to respiratory compromise and secondary hypoxic–ischemic encephalopathy, offering an alternative hypothesis for some intracranial findings traditionally attributed to primary intracranial trauma. Importantly, their study emphasized that such cervical nerve root injuries are not detectable with routine autopsy techniques and require targeted dissection and histologic examination of the cervical spine and associated soft tissues.

In the present series, cervical spinal cord and meningeal hemorrhages were identified in a substantial proportion of cases, supporting the value of careful cervical spine examination in pediatric head trauma. Cervical spinal cord involvement was observed in 41.1% of cases, with extradural and subdural hemorrhages being the most frequent lesions. The frequency and anatomical distribution of cervical spinal cord and meningeal hemorrhages observed in this series highlight the potential for clinically and forensically relevant findings in regions that may be incompletely assessed with routine autopsy techniques. In particular, hemorrhagic changes involving the cervical spinal cord, nerve roots, and adjacent soft tissues may be subtle, anatomically complex, and easily overlooked when examination is limited or fragmented.

Despite increasing recognition of spinal cord involvement in abusive head trauma, examination of the cervical spine remains non standardized in many forensic protocols. Conventional autopsy techniques often involve separate removal of the brain and spinal cord, which may limit evaluation of the cervicomedullary region. To address these limitations, several extended dissection techniques have been described, including immersion methods for preservation of fragile brain tissue, posterior approaches allowing continuous removal of the brain and spinal cord, and en bloc cervical spine techniques that preserve anatomical relationships among the spinal cord, nerve roots, ganglia, and surrounding soft tissues [56–63]. While these approaches may enhance visualization of deep cervical structures, they are technically demanding and their applicability depends on available resources and expertise, particularly in infant autopsies.

**Fig. 3:**
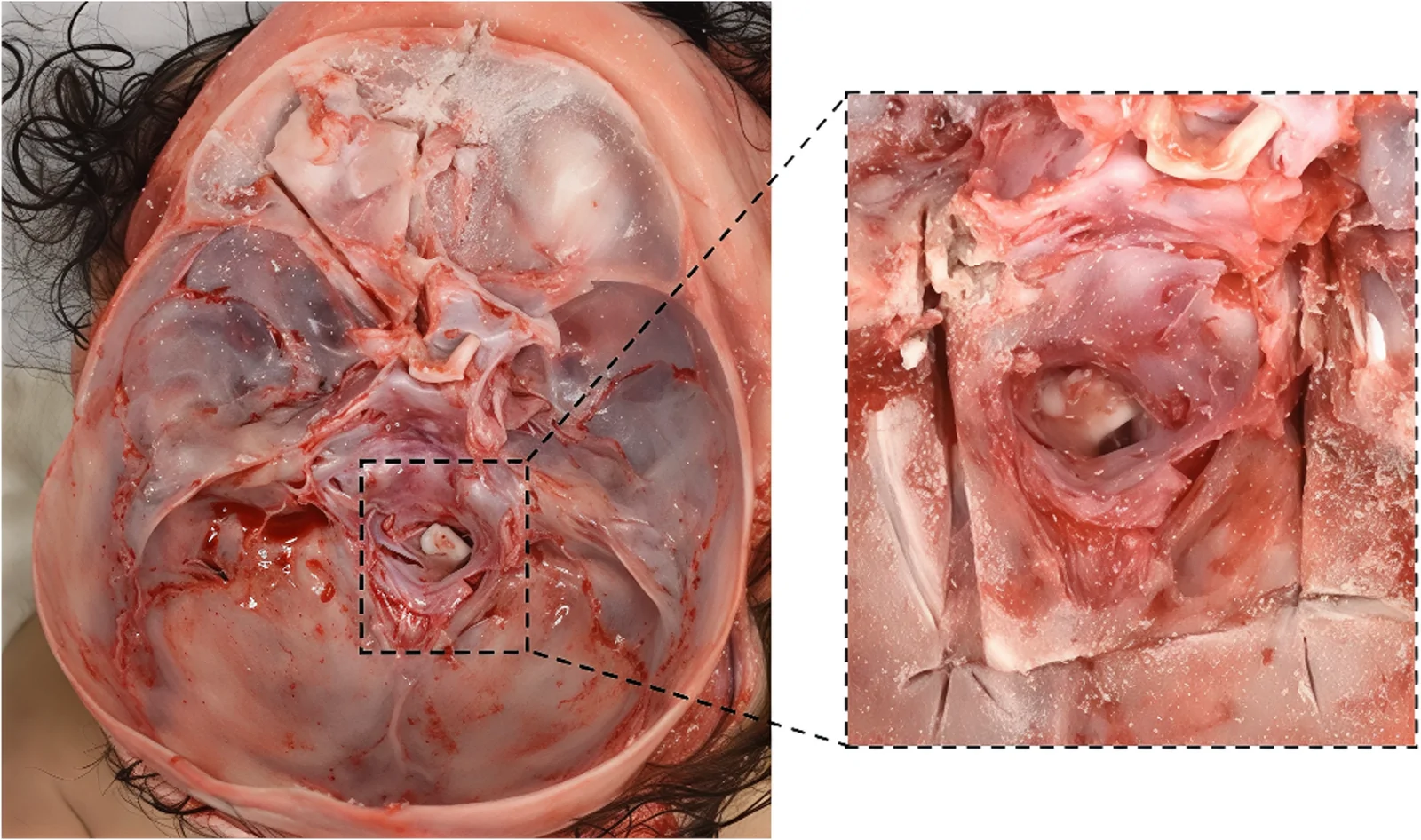
Cranial base showing the periforamen magnum region. Approximate landmarks are shown on the left, and the final result after dissection with an oscillating blade is shown on the right

**Fig. 4:**
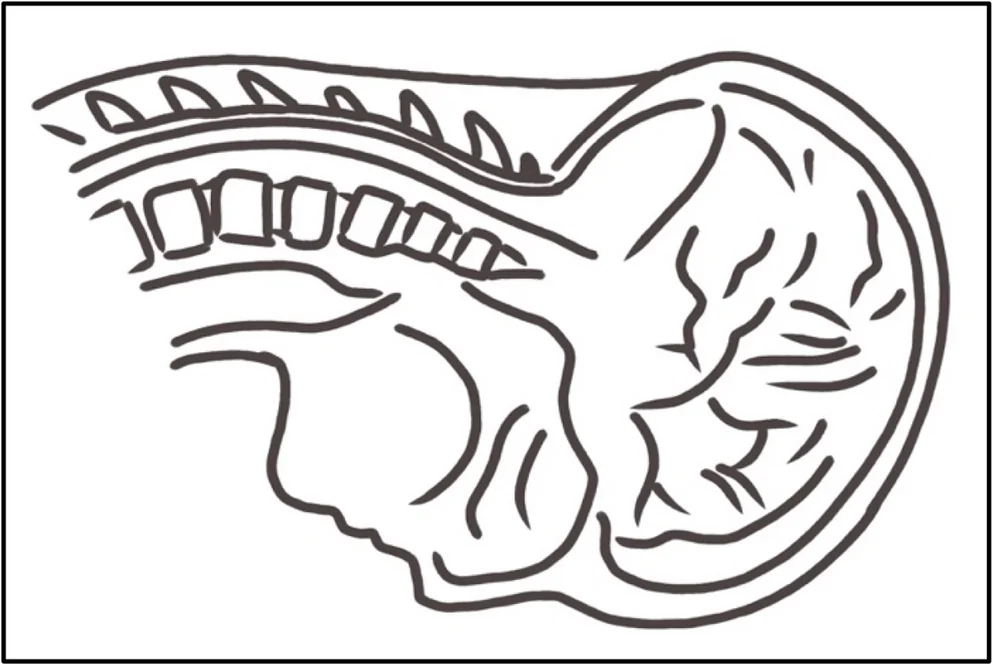
Hyperflexion of the neck

**Fig. 5:**
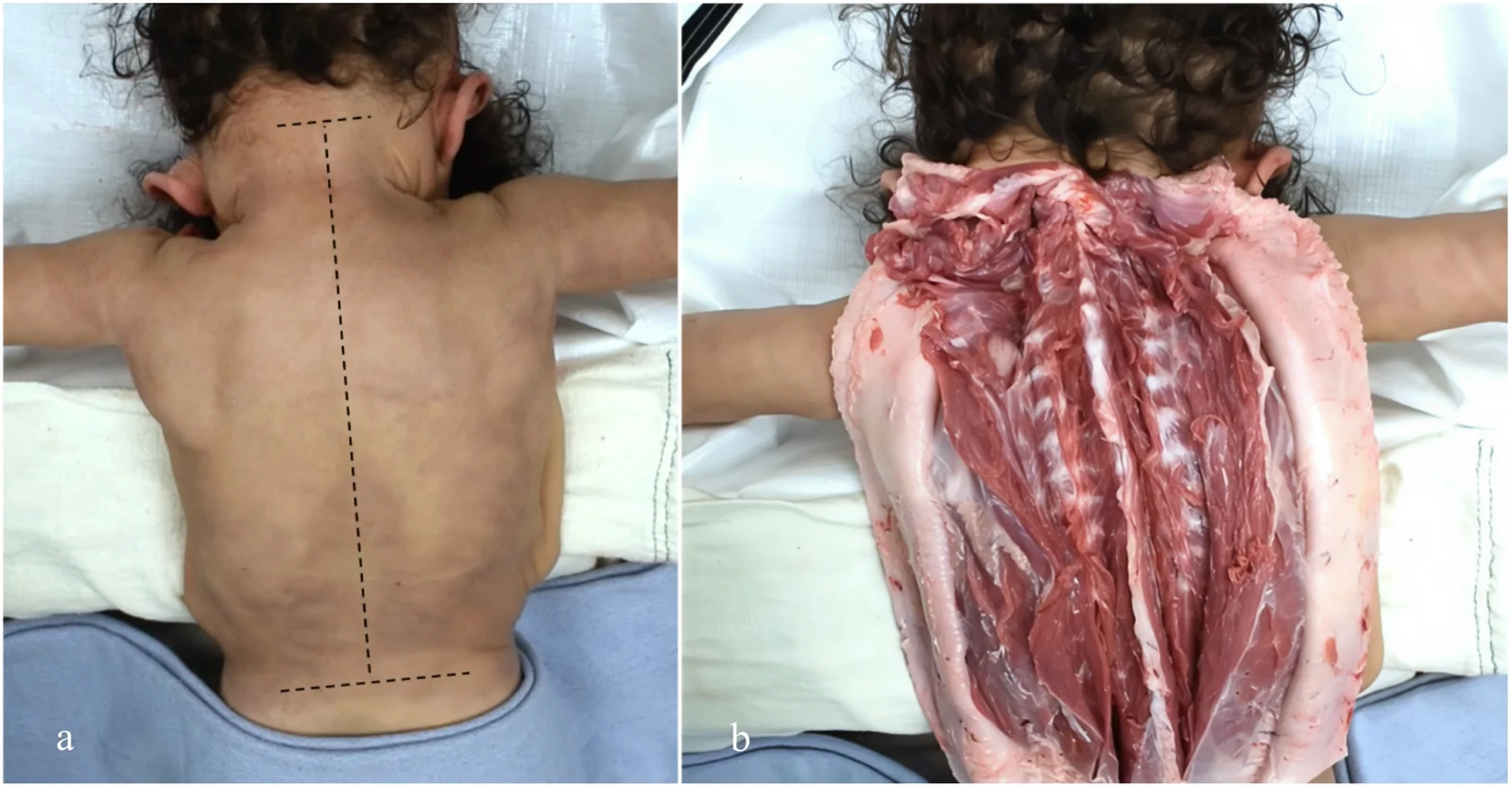
(**a**) Dissection landmarks for the posterior body dissection. (**b**) Posterior body skin and soft tissues dissection

**Fig. 6:**
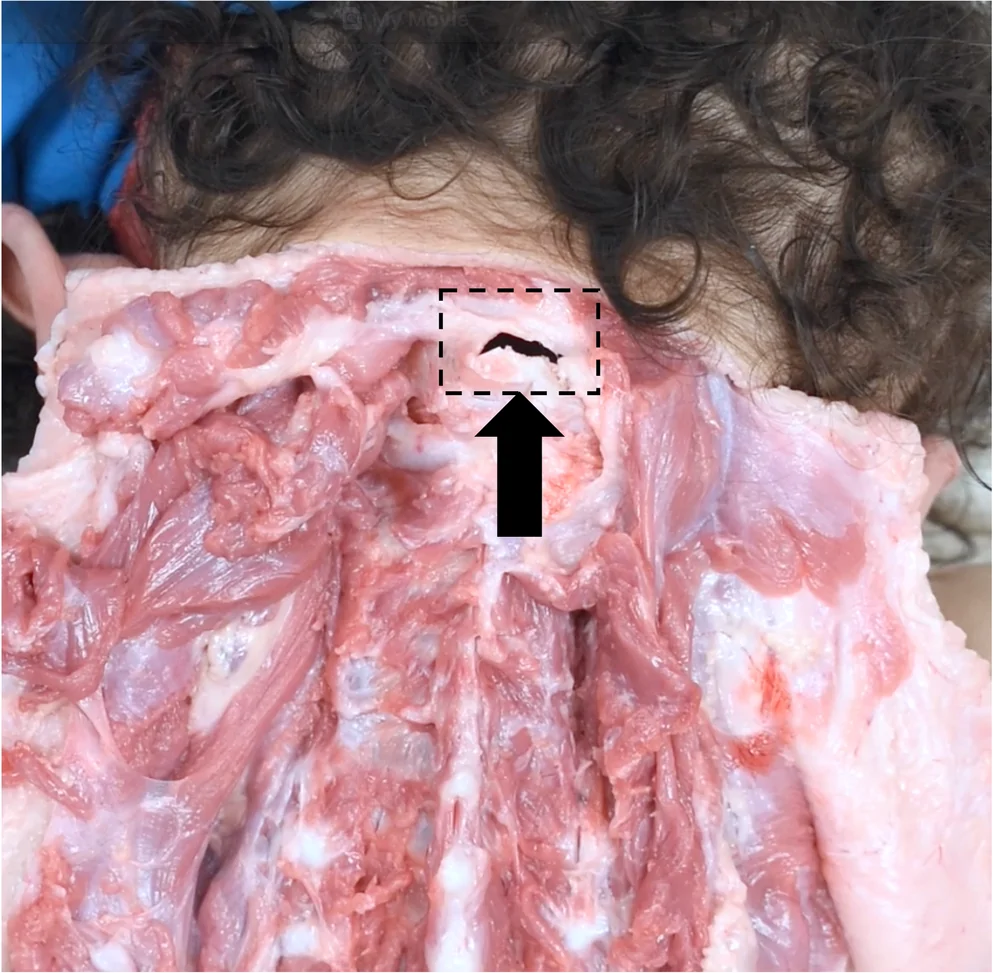
Area of tissues discontinuation between the proximal aspect of the cervical spine and the skull base (black arrow and dotted square)

For this reason, the authors of this study propose a slight revision of the method described by Ali et al. [63], employing a primarily posterior approach to the cervical spine rather than the anterior approach originally described. This alternative technique provides broader anatomical exposure and facilitates preservation of relevant structures for documentation. The dissection technique for en bloc removal of the cervical spine and spinal cord following the posterior approach involves the following steps:

1. *Preparation*: With the body placed supine, a complete autopsy is performed, including removal of the thoracic and abdominal organs, skull opening and brain removal to expose the anterior, middle, and posterior cranial fossae, and the layered anterior neck dissection. The distal portions of carotid arteries are delicately detached from the surrounding neck tissues and moved away from the spine.
2. *Occipital Bone Section*: From the intracranial cavity, the periforamen magnum region of the occipital bone is sawed using an oscillating saw to create a square or rectangular cut around the foramen magnum (Fig. 3). Suggested dissection landmarks include: a superior border at the level of the sella turcica, an inferior border approximately 2 cm from the posterior margin of the foramen magnum, and lateral borders approximately 1–2 cm from the lateral margins of the foramen. The cut should be deep enough to allow for the visualization of the external soft tissues of the skull base and posterior neck.
3. *Change of body position*: The body is placed in the prone position, with a support block positioned beneath the chest to allow the cervical region to become naturally hyperflexed (Fig. 4).
4. *Posterior dissection*: A posterior body dissection is performed to expose the soft tissues and spine, extending superiorly to visualize the base of the skull and the foramen magnum (Fig. 5a, b). If hemorrhage of the subcutaneous tissues or skeletal muscles is observed, a layered posterior skin dissection should be conducted.
5. *Cervical Spine Isolation*: If the periforamen magnum segment has been sawed correctly, the operator should be able to identify a discontinuity between the proximal portion of the cervical spine and the base of the skull (Fig. 6).

**Fig. 7:**
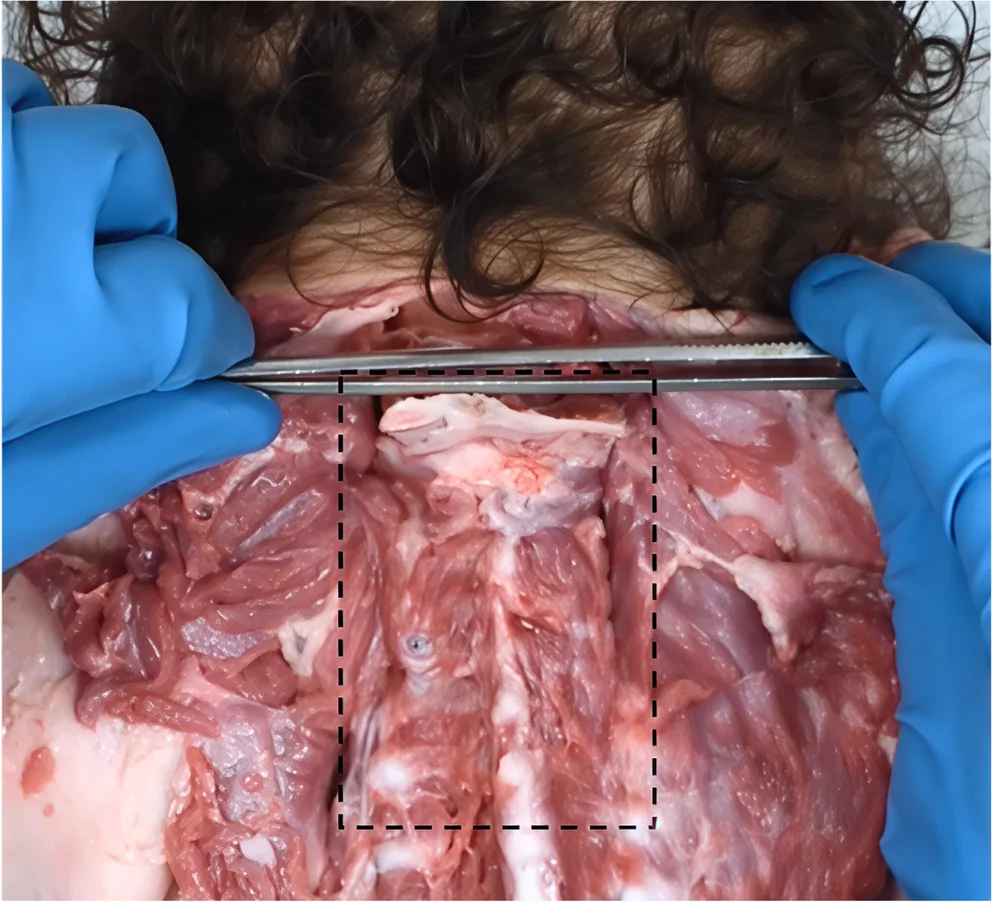
Dissection landmarks for the isolation and removal of cervical spine block

The residual soft tissue bands are cleared using a sharp scalpel. The soft tissues surrounding the cervical spine are then dissected, and a transverse incision is made between C7 and T1 to isolate the cervical region (Fig. 7).

Using an oscillating saw, longitudinal cuts are made through the first bilateral ribs along the paravertebral regions, approximately 2 cm distal to the costovertebral junction. This facilitates the safe preservation of the nerve root ganglia. Once the remaining regional soft tissues are cleared, the cervical spine, including the spinal cord and associated nerve root ganglia, can be removed en bloc.

**Fig. 8:**
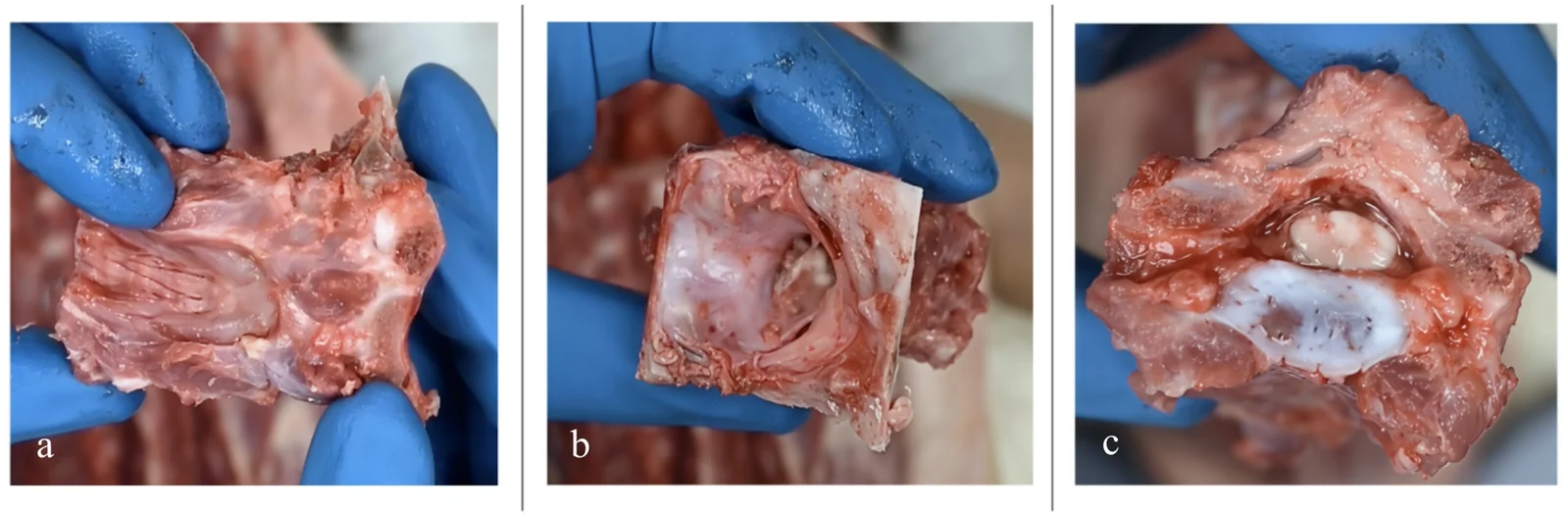
Cervical block. (**a**) Lateral view; (**b**) superior view; (**c**) inferior view

6. *Retaining the cervical spine block*. The extracted cervical spine segment (Fig. 8a-c) is then fixed in formaldehyde, decalcified, serially sectioned, and examined both grossly and microscopically.

Forensic pathologists should be aware that this method can be resource-intensive and is best reserved for suspected trauma or homicide cases rather than routine autopsies [62]. This technique necessitates adjustments in the histology laboratory, including the implementation of new protocols and the use of specialized equipment to accommodate the processing and staining of larger tissue blocks. Nevertheless, once mastered, the technique enables rapid and efficient evisceration of the entire cervical spine block and is applicable to pediatric subjects of any age.

Finally, ancillary studies, such as postmortem toxicology, microbiology (including blood and tissue cultures, and viral studies – to be collected at the beginning of the internal examination to avoid contaminations), and carbon monoxide testing, are also essential in suspected child abuse cases, even when the cause of death appears to be obvious trauma. These investigations help rule out natural disease processes, identify coexisting conditions (e.g., infections or metabolic disorders), detect substances that may increase the child’s vulnerability, and exclude alternative explanations for clinical or autopsy findings.

## Conclusion

In pediatric non accidental injury cases, postmortem evaluation is often challenging, particularly in infants in whom external signs of trauma may be limited. This study documents the distribution of intracranial, ocular, spinal, and extracranial findings observed in infants with homicidal blunt head trauma and highlights the frequent involvement of the cervical spinal cord and associated meningeal structures.

The observed cervical spinal findings underscore the importance of careful examination of the cervicomedullary region during forensic autopsy. Although these findings are not diagnostic in isolation, their documentation may contribute to a more complete anatomical assessment when interpreted in conjunction with intracranial and extracranial injuries.

The en bloc cervical spine dissection technique described is presented as a technical option to enhance visualization and preservation of cervical structures in selected cases. Its use requires appropriate expertise and resources and should be guided by case specific considerations rather than applied routinely.

It is critical to recognize that autopsy findings alone can only support a thorough medicolegal death investigation and should not be used in isolation to diagnose inflicted head trauma in children. Comprehensive and well documented postmortem examinations, including appropriate ancillary studies, are essential to address alternative explanations and to clearly define anatomical findings in complex pediatric deaths, supporting scientific transparency and clarity in medicolegal proceedings.

## Data Availability

Data for this research is easily accessible through the International Journal of Legal Medicine website.
